# Real-World Outcomes of Fusion-Directed Targeted Therapy in Advanced Non-Small Cell Lung Cancer Harboring Actionable Gene Fusions

**DOI:** 10.3390/ijms27177849

**Published:** 2026-09-02

**Authors:** Faure Delgado Leon, Suset Almuinas de Armas, Melanie Molina, Eric G. Morales, Maria Fernandez-Gomez, Luis Estuardo Raez

**Affiliations:** 1 Division of Internal Medicine, Memorial Healthcare System, 703 N Flamingo Road, Pembroke Pines, FL 33028, USA; 2 Charles E. Schmidt College of Medicine, Florida Atlantic University, Boca Raton, FL 33431, USA; 3 Oncology Research Department, Memorial Cancer Institute, Memorial Healthcare System, Pembroke Pines, FL 33026, USA; 4 Memorial Cancer Institute, Memorial Healthcare System, 703 N Flamingo Road, Pembroke Pines, FL 33028, USA

**Keywords:** non-small cell lung cancer, gene fusion, targeted therapy, precision oncology, next-generation sequencing, real-world evidence

## Abstract

Actionable ALK, ROS1, RET, NTRK, and NRG1 fusions define biologically distinct subsets of advanced non-small cell lung cancer (NSCLC), yet real-world data across these rare populations remain limited. We retrospectively evaluated patients with advanced NSCLC treated at Memorial Healthcare System who received genotype-matched fusion-directed therapy; the first fusion-directed agent defined the index treatment, and survival was measured from its initiation. Progression was determined from radiographic reports and treating-oncologist documentation. After patient-level reconciliation, 59 unique patients were included: 29 ALK, 14 ROS1, 11 RET, 3 NTRK, and 2 NRG1. Median follow-up was 47.7 months (95% CI, 28.6–58.7), median progression-free survival was 44.8 months (95% CI, 17.1–not estimable), and median overall survival was not reached. No statistically significant survival differences were detected among ALK, ROS1, and RET subgroups, although limited sample sizes preclude exclusion of clinically meaningful differences. Later-line index therapy was not significantly associated with progression-free survival in exploratory unadjusted analysis. These findings provide descriptive real-world evidence of precision-oncology implementation across actionable fusion-defined NSCLC while underscoring that pooled outcomes should not be interpreted as evidence of equivalent efficacy across molecular subtypes or therapies.

## 1. Introduction

Lung cancer remains the leading cause of cancer-related mortality worldwide, accounting for approximately 2.5 million new cases and 1.8 million deaths in 2022. Non-small cell lung cancer (NSCLC) comprises approximately 85% of lung cancers and remains a major global health burden. Over the past two decades, management of advanced NSCLC has shifted from a predominantly histology-based approach toward precision oncology, driven by identification of actionable molecular alterations and development of targeted therapies. Comprehensive molecular profiling by next-generation sequencing (NGS) is recommended for patients with advanced non-squamous NSCLC because identification of actionable alterations directly informs treatment selection [1,2,3,4].

Among the most clinically relevant genomic alterations are oncogenic gene fusions, which arise through chromosomal rearrangements that juxtapose previously separate genes and may produce constitutively active fusion proteins. The principal actionable fusion groups in NSCLC include ALK, ROS1, RET, NTRK1/2/3, and NRG1. Although each is individually uncommon, together they identify a clinically important population eligible for fusion-directed therapy [2,5,6].

The therapeutic landscape for fusion-positive NSCLC has evolved rapidly. Primary studies of next-generation ALK inhibitors, ROS1 inhibitors, selective RET inhibitors, tumor-agnostic TRK inhibitors, and zenocutuzumab for NRG1 fusion-positive cancer have established substantial and often durable antitumor activity across these molecular subsets [7,8,9,10,11,12,13,14,15,16].

The widespread implementation of comprehensive genomic profiling has changed the diagnostic and therapeutic paradigm of advanced NSCLC. Contemporary NGS platforms can evaluate multiple cancer-associated genes simultaneously, improving the ability to identify both common and rare actionable alterations from limited specimens. Molecular-testing recommendations therefore support broad profiling before systemic treatment in advanced non-squamous NSCLC.

Despite these advances, important knowledge gaps remain. Most efficacy evidence originates from small prospective trials with protocol-defined eligibility, whereas real-world investigations have generally focused on individual fusion subtypes or biomarker prevalence. Few cohorts have applied common outcome definitions across all currently actionable fusion groups within one health system [17,18,19,20].

We therefore conducted a retrospective health-system cohort study to characterize the clinicopathologic features, treatment patterns, and survival outcomes of patients with advanced NSCLC harboring ALK, ROS1, RET, NTRK, or NRG1 gene fusions who received fusion-directed targeted therapy. The rationale for evaluating these alterations within a common cohort was clinical rather than biological: each fusion defines a distinct molecular disease with different signaling biology, targeted inhibitors, and resistance mechanisms, and therapeutic equivalence was not assumed. The pooled analysis was designed to assess real-world implementation of genotype-matched fusion-directed therapy within a single health system using uniform eligibility criteria, outcome definitions, and follow-up methodology, while fusion-specific analyses were used to examine the larger ALK, ROS1, and RET subgroups. We hypothesized that patients treated in routine clinical practice would experience durable disease control despite the greater clinical heterogeneity inherent to a real-world population.

## 2. Results

### 2.1. Patient Cohort

Between 14 May 2014, and 10 October 2025, 59 unique patients with advanced lung adenocarcinoma harboring an actionable gene fusion met the analytic eligibility criteria and received an index fusion-directed targeted therapy at Memorial Cancer Institute. During patient-level audit, sequential targeted-therapy records belonging to the same patient were collapsed to the first qualifying fusion-directed therapy in accordance with the prespecified index-treatment definition. One additional patient with an ALK rearrangement and concurrent EGFR alteration was excluded because the recorded treatment was osimertinib, which is EGFR-directed and did not meet the fusion-directed index-treatment definition. Clinical follow-up continued through 24 April 2026. Median follow-up by reverse Kaplan-Meier analysis was 47.7 months (95% CI, 28.6–58.7 months). A separate pre-database screening log was unavailable; therefore, the total number initially screened and individual exclusion counts before creation of the analytic dataset could not be reliably reconstructed.

### 2.2. Clinicopathologic Characteristics

The median age at cancer diagnosis was 63 years (range, 27–90 years); 35 patients (59.3%) were female, and 29 (49.2%) were never-smokers. All tumors were adenocarcinomas. Race was recorded as White in 51 patients (86.4%), Black in 6 (10.2%), and Asian in 2 (3.4%). Hispanic ethnicity was explicitly documented in 36 patients (61.0%); the remaining 23 records contained non-Hispanic or other unstructured demographic descriptors and were not interpreted as mutually exclusive ethnicity categories. (Table 1).

### 2.3. Molecular Landscape and Treatment Patterns

ALK rearrangements were the most common actionable alteration (29 patients, 49.2%), followed by ROS1 (14, 23.7%), RET (11, 18.6%), NTRK (3, 5.1%), and NRG1 (2, 3.4%). Fusion-directed index therapy was administered in the first-line setting in 39 patients (66.1%), second-line in 15 (25.4%), and third-line in 5 (8.5%). Alectinib was the most frequently used ALK-directed index therapy, entrectinib the most frequently used ROS1-directed therapy, selpercatinib the predominant RET-directed therapy, larotrectinib was the index therapy for all three NTRK cases, and zenocutuzumab was used in both NRG1 cases (Table 2).

### 2.4. Survival Outcomes

**Figure 1:**
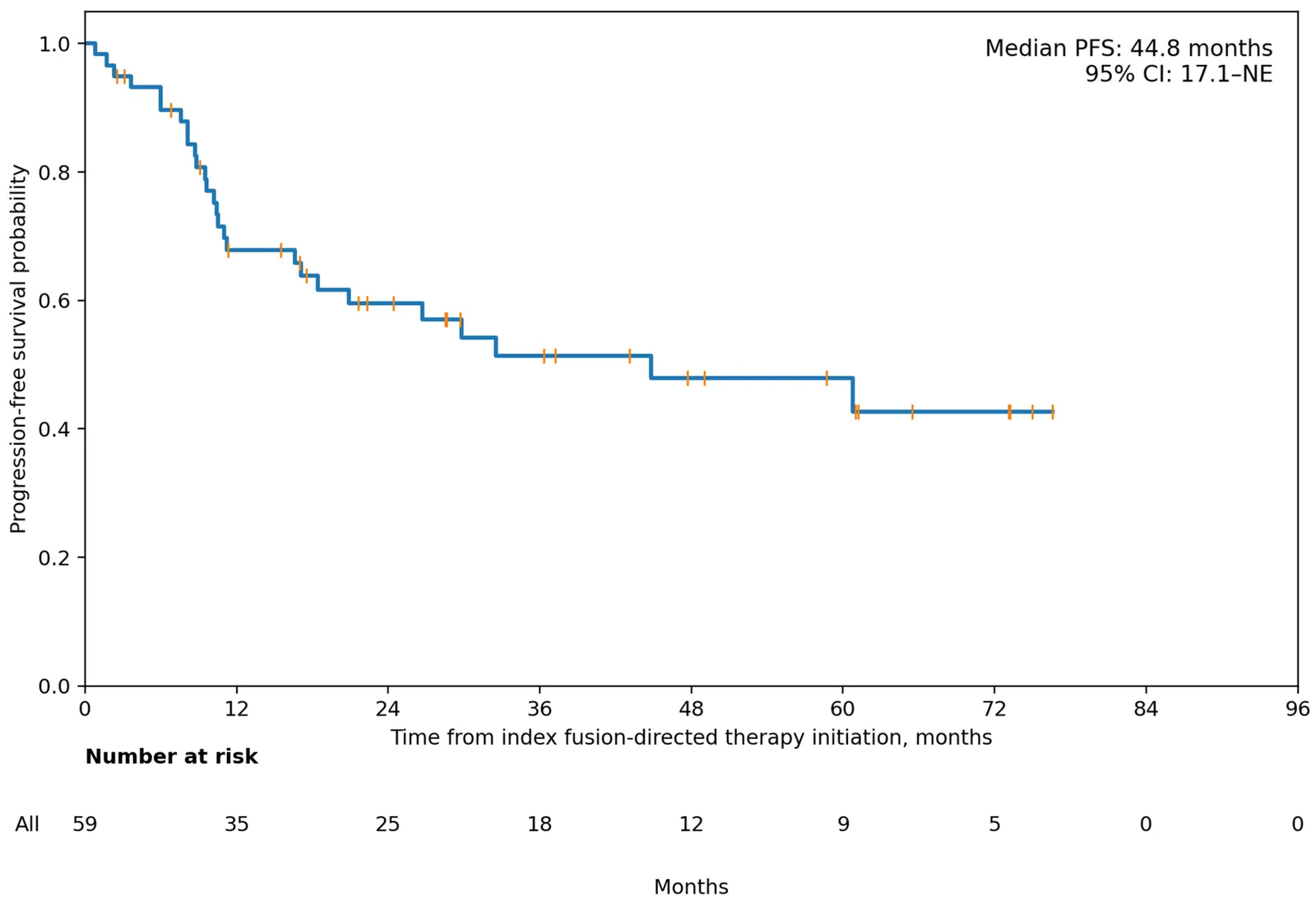
Progression-free survival in the overall analytic cohort. Kaplan-Meier estimate from initiation of the first fusion-directed targeted therapy among 59 unique patients. Median PFS was 44.8 months (95% CI, 17.1 months to not estimable). Vertical tick marks indicate censored observations. Numbers at risk are shown below the plot; late estimates should be interpreted cautiously as the risk set becomes small. CI, confidence interval; NE, not estimable; PFS, progression-free survival.

**Figure 2:**
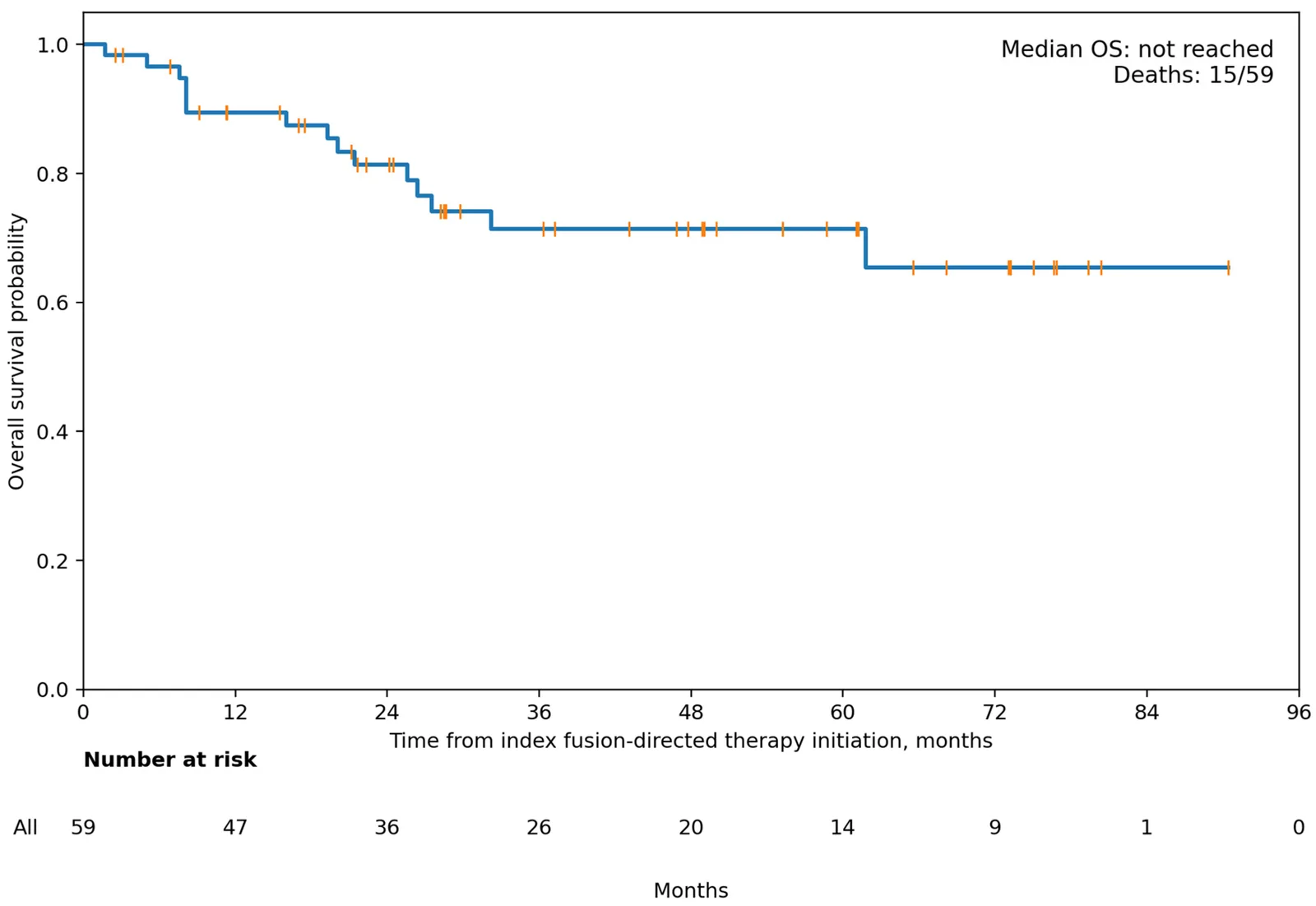
Overall survival in the overall analytic cohort. Kaplan-Meier estimate from initiation of the first fusion-directed targeted therapy among 59 unique patients. Median OS was not reached; 15 deaths and 44 censored observations were recorded by 24 April 2026. Vertical tick marks indicate censored observations. Numbers at risk are shown below the plot; late estimates should be interpreted cautiously as the risk set becomes small. OS, overall survival.

At the data cutoff, 27 PFS events had occurred and 32 patients (54.2%) were censored for PFS. Fifteen deaths had occurred, and 44 patients (74.6%) were censored for OS. Median PFS for the pooled cohort was 44.8 months (95% CI, 17.1 months to not estimable), whereas median OS was not reached. Estimated PFS rates at 12, 24, and 36 months were 67.8% (95% CI, 53.9–78.4%), 59.5% (95% CI, 45.1–71.3%), and 51.4% (95% CI, 36.4–64.5%), respectively. Corresponding OS estimates were 89.4% (95% CI, 77.9–95.1%), 81.3% (95% CI, 67.9–89.5%), and 71.4% (95% CI, 56.1–82.2%). These pooled estimates are descriptive summaries of a heterogeneous cohort rather than a general treatment-effect estimate. Kaplan-Meier curves for the overall cohort are shown in Figure 1 and Figure 2.

### 2.5. Survival by Fusion Subtype

**Figure 3:**
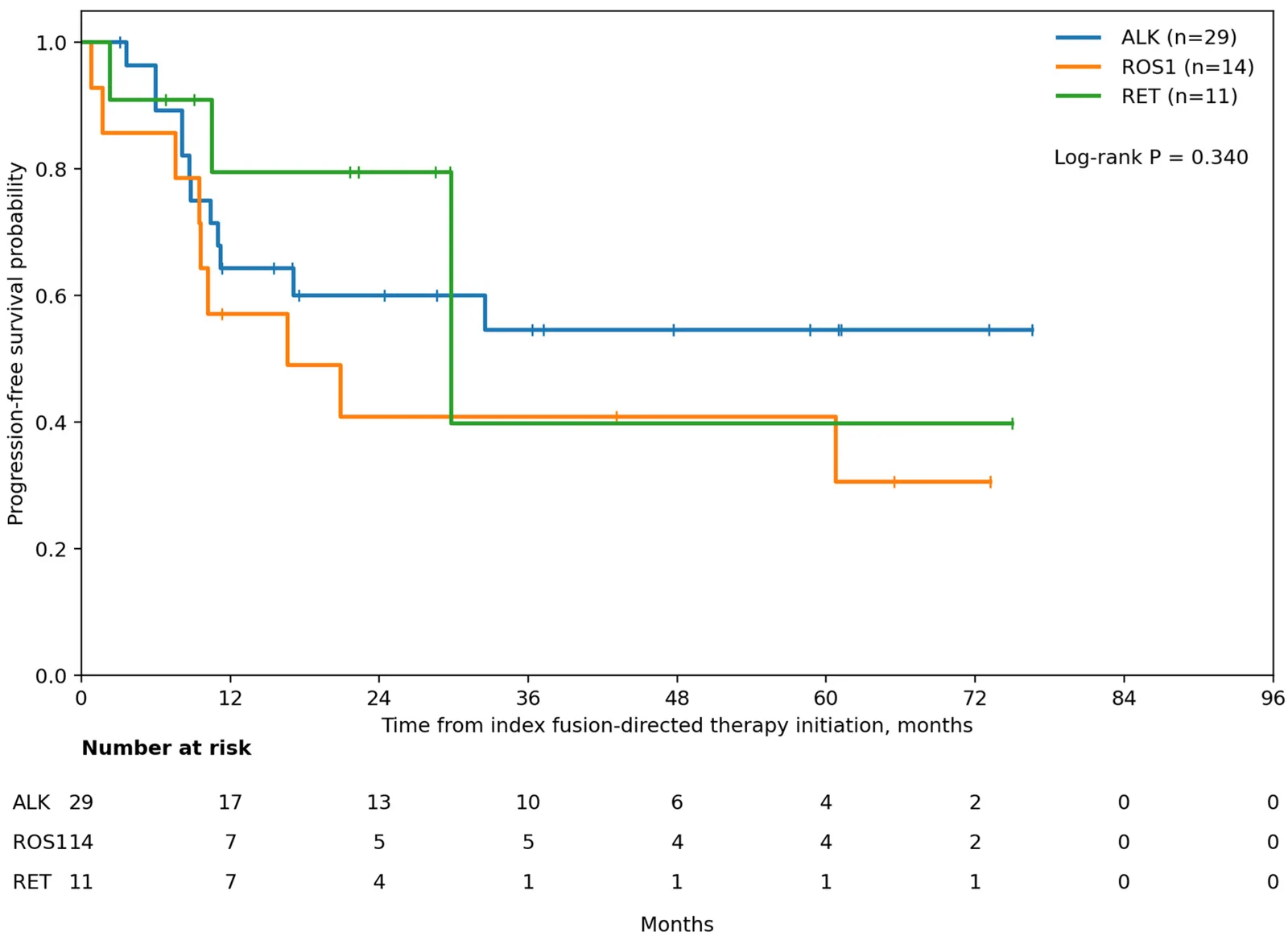
Progression-free survival according to fusion subtype. Kaplan-Meier estimates for ALK (n = 29), ROS1 (n = 14), and RET (n = 11). NTRK and NRG1 were not plotted because of small subgroup sizes. No statistically significant difference in PFS distributions was detected among the three plotted subgroups (log-rank *p* = 0.340); the comparison was underpowered and clinically meaningful differences cannot be excluded. Vertical tick marks indicate censored observations. Numbers at risk are shown below the plot. PFS, progression-free survival.

Among the three largest molecular subgroups, median PFS was not reached for ALK, 16.6 months for ROS1, and 29.8 months for RET. Median OS was not reached for ALK and ROS1 and was 61.8 months for RET. No statistically significant differences in survival distributions were detected among ALK, ROS1, and RET for PFS (log-rank *p* = 0.340) or OS (log-rank *p* = 0.948). These comparisons were underpowered, particularly for RET, which had only three PFS events; therefore, clinically meaningful differences cannot be excluded. Estimates for NTRK and NRG1 were descriptive because of small subgroup sizes (Table 3). Kaplan-Meier estimates of PFS by fusion subtype are presented in Figure 3.

### 2.6. Exploratory Analysis by Treatment Line

**Figure 4:**
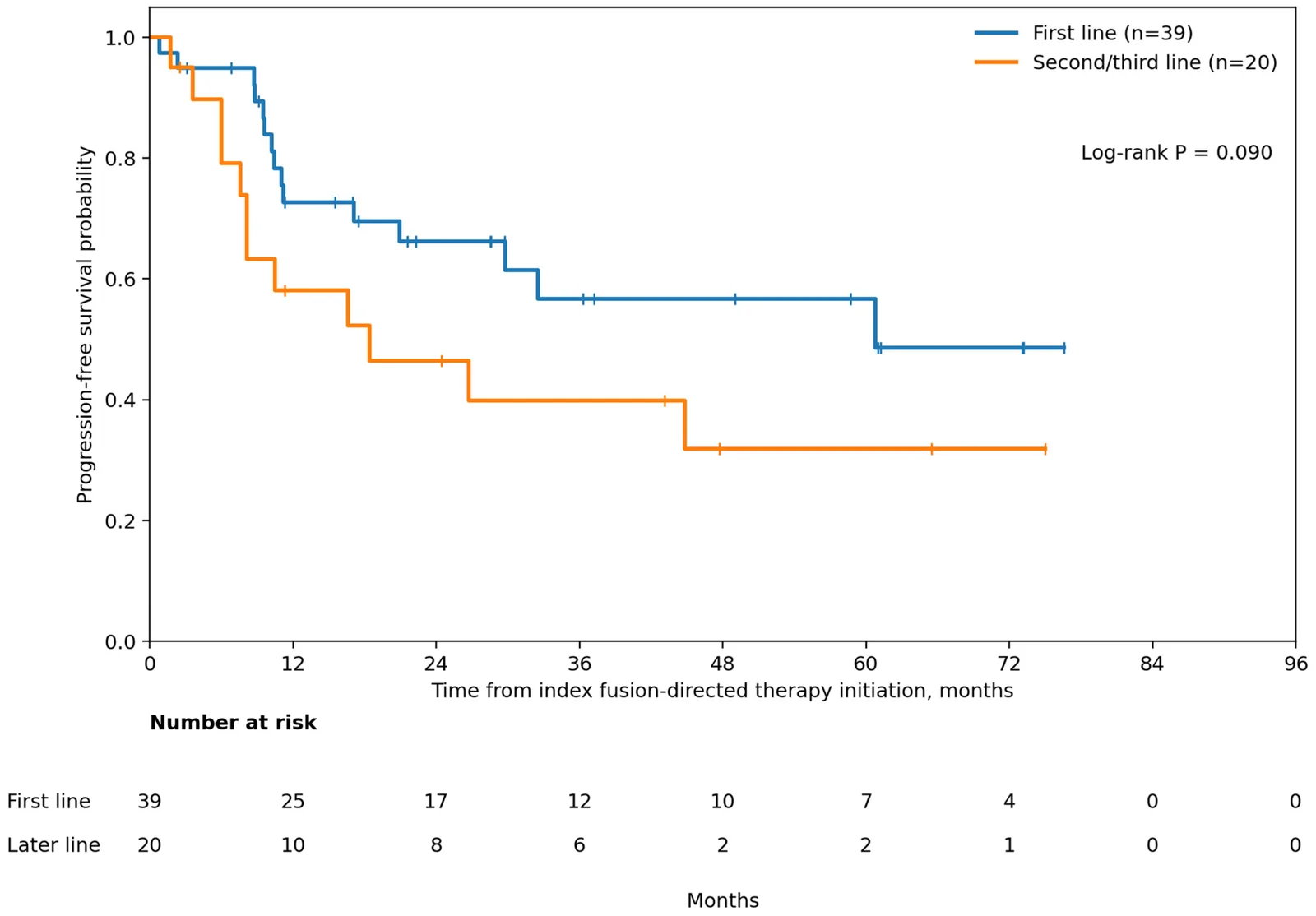
Progression-free survival according to targeted-therapy line. Kaplan-Meier estimates for first-line index therapy (n = 39) versus second/third-line index therapy (n = 20). The PFS distributions were not statistically significantly different (log-rank *p* = 0.090). This comparison was exploratory, unadjusted, and should not be interpreted causally. Vertical tick marks indicate censored observations. Numbers at risk are shown below the plot. PFS, progression-free survival.

Median PFS was 60.8 months among patients whose index fusion-directed therapy was first line and 18.4 months among those whose index therapy was second or third line; the difference was not statistically significant (log-rank *p* = 0.090). Median OS was not reached in either group, although the OS distributions differed in this unadjusted comparison (log-rank *p* = 0.038). There were 15 PFS events among 39 first-line patients and 12 among 20 later-line patients; deaths occurred in 7 of 39 first-line patients and 8 of 20 later-line patients. In exploratory univariable Cox models, later-line index therapy was not significantly associated with progression or death (HR, 1.92; 95% CI, 0.90–4.10; *p* = 0.094), while a higher hazard of death was observed (HR, 2.83; 95% CI, 1.02–7.83; *p* = 0.045). These unadjusted associations are strongly susceptible to confounding by treatment era, fusion subtype, drug generation, prior systemic therapy, performance status, disease burden, brain metastases, and access to molecular testing, many of which were not available for adjustment. The findings should therefore not be interpreted as evidence that earlier administration independently improves survival (Table 4; Figure 4).

## 3. Discussion

In this retrospective cohort of 59 unique patients with advanced lung adenocarcinoma harboring actionable gene fusions, genotype-matched fusion-directed targeted therapy was associated with durable disease control in routine clinical practice. The pooled median PFS was 44.8 months, and median OS was not reached after a reverse Kaplan-Meier median follow-up of 47.7 months. These values summarize a heterogeneous health-system cohort and should not be interpreted as a general measure of the effectiveness of fusion-directed therapy. No statistically significant differences in survival distributions were detected among ALK, ROS1, and RET, but the study was underpowered for comparative inference and clinically important differences cannot be excluded. The exploratory treatment-line analysis did not detect a statistically significant association with PFS; the observed OS association was unadjusted and strongly confounded and should not be interpreted as evidence that first-line administration independently improves survival.

Importantly, the pooled cohort should not be interpreted as a biologically homogeneous disease group or as evidence that ALK-, ROS1-, RET-, NTRK-, and NRG1-driven tumors have equivalent sensitivity to targeted therapy. These alterations differ in oncogenic signaling, fusion partners, approved inhibitors, resistance mechanisms, treatment era, and the evidence supporting individual agents. The pooled estimates instead summarize health-system-level outcomes after genotype-matched fusion-directed therapy under common outcome definitions. For this reason, fusion-specific estimates are clinically more informative when subgroup size permits, whereas the pooled analysis addresses the implementation of precision oncology across actionable fusion-positive NSCLC.

### 3.1. Contextual Comparison with Landmark Trials

Selected prospective trials are discussed only to provide molecular-subtype-specific clinical context, not as comparators for the present observational cohort. Differences in eligibility, drug generation, treatment line, endpoint ascertainment, imaging schedules, and follow-up preclude direct efficacy comparisons. In ALK-positive disease, the updated ALEX analysis reported a median investigator-assessed PFS of 34.8 months with first-line alectinib, whereas the 7-year CROWN update reported that median PFS with lorlatinib remained unreached and approximately 55% of patients remained progression-free at 7 years. In our smaller ALK subgroup, median PFS and OS were not reached; this finding should be interpreted descriptively and not as evidence of equivalence to prospective trials.

### 3.2. ROS1-Positive Disease

For ROS1 fusion-positive NSCLC, the updated PROFILE 1001 analysis reported median PFS of 19.3 months and median OS of 51.4 months with crizotinib [9]. A long-term integrated analysis of entrectinib reported median PFS of 15.7 months and median OS of 47.8 months, whereas TRIDENT-1 reported median PFS of 35.7 months among ROS1 TKI-naive patients treated with repotrectinib. The median PFS of 16.6 months in our ROS1 subgroup is therefore most similar to the crizotinib and entrectinib experience, which is clinically plausible given the mixture of agents, treatment eras, and treatment lines represented in this cohort.

### 3.3. RET Fusion-Positive Disease

In RET fusion-positive NSCLC, the updated LIBRETTO-001 analysis reported durable activity with selpercatinib, including median PFS of 24.9 months in the efficacy population [12]. In the randomized LIBRETTO-431 trial, first-line selpercatinib produced median PFS of 24.8 months and an objective response rate of 84% [13]. The updated ARROW analysis reported an objective response rate of 72% and median PFS of 13.0 months among treatment-naive patients receiving pralsetinib. Median PFS in our RET subgroup was 29.8 months and median OS was 61.8 months. These results are directionally consistent with sustained benefit from selective RET inhibition, but the small number of RET patients and events precludes definitive comparison.

### 3.4. Rare NTRK and NRG1 Fusions

The NTRK and NRG1 cohorts were too small for meaningful survival inference, but their inclusion illustrates the clinical value of broad fusion testing. In a lung cancer-specific pooled analysis, larotrectinib produced an objective response rate of 73%, median PFS of 35.4 months, and median OS of 40.7 months in evaluable patients with TRK fusion-positive lung cancer. In the eNRGy trial, zenocutuzumab demonstrated activity in NRG1 fusion-positive cancer, including NSCLC [16]. Our three unique NTRK-positive and two NRG1-positive patients provide descriptive real-world evidence only and should not be compared numerically with these prospective cohorts. Among the NTRK cases, one was specifically documented as NTRK1, whereas the other two lacked sufficient gene-level annotation to distinguish NTRK1, NTRK2, or NTRK3.

### 3.5. Real-World Evidence and Study Contribution

Existing real-world studies have generally examined individual fusion subtypes, biomarker prevalence, or specific registries [17,18,19,20]. The contribution of the present study is narrower: it describes the implementation and outcomes of genotype-matched therapy across five actionable fusion-defined NSCLC populations within one healthcare system using common index-date and survival definitions, including rare NTRK and NRG1 cases. This unified framework allows consistent descriptive reporting across the health system, but the sample size does not support definitive comparative-effectiveness claims among fusion subtypes, drugs, or treatment sequences.

The relatively high proportion of patients with explicitly documented Hispanic ethnicity (61.0%) likely reflects the demographic composition and referral patterns of the South Florida population served by Memorial Healthcare System rather than intentional enrichment. Memorial Healthcare System is located in Broward County, where approximately 35.0% of residents identify as Hispanic or Latino, and the broader South Florida region includes neighboring Miami-Dade County, where approximately 70.6% identify as Hispanic or Latino [21,22]. Because this was a single-health-system retrospective cohort and demographic information was abstracted from the electronic health record, the observed proportion should not be interpreted as a population-based estimate of fusion prevalence or treatment outcomes by ethnicity.

### 3.6. Precision Oncology in Routine Practice

These findings support comprehensive genomic profiling as a prerequisite for precision treatment in advanced nonsquamous NSCLC. Broad molecular testing can identify both common and rare actionable rearrangements before systemic treatment decisions are finalized. The treatment-line analysis should be viewed only as hypothesis-generating because line of therapy was correlated with treatment era and prior treatment exposure and could also reflect unmeasured differences in performance status, disease burden, brain metastases, fusion subtype, drug generation, and access to testing.

### 3.7. Implications for Immunotherapy

The study did not directly evaluate immunotherapy efficacy. Nevertheless, systematic reviews and meta-analyses have generally reported limited activity of immune checkpoint inhibitors in oncogene-driven NSCLC, including RET fusion-positive and other driver-defined populations [23,24,25]. These data support obtaining molecular results before systemic treatment whenever clinically feasible and prioritizing an approved targeted therapy when a relevant actionable fusion is identified.

### 3.8. Strengths and Limitations

This study has several strengths, including patient-level outcome data, reverse Kaplan-Meier follow-up approaching 4 years, inclusion of five clinically actionable fusion groups, and uniform index-date and survival definitions within one health system. Important limitations substantially constrain comparative interpretation. The study was retrospective, single-system, modest in size, and heterogeneous across an 11-year diagnostic period, molecular subtypes, targeted agents, drug generations, and treatment lines. Only 27 PFS events and 15 deaths occurred. ECOG performance status, brain metastases, disease burden, comorbidities, detailed prior systemic treatments, treatment duration, discontinuation reasons, dose modifications, subsequent therapies, and consistently structured fusion-partner/variant data were not available in the locked analytic dataset and could not be recovered reliably for this revision. Individual-patient molecular-testing platform, specimen source, and assay-level DNA/RNA methodology were also not systematically retained. Progression ascertainment relied on routine radiology reports and treating-oncologist documentation rather than central RECIST review, and imaging intervals were not protocol mandated. Demographic source fields contained unstructured descriptors of ethnicity, nationality, geographic origin, and cultural identity and therefore were not used as mutually comparable ethnicity categories. The unadjusted treatment-line models could not account for major prognostic and treatment-selection confounders. Finally, pooled and cross-trial estimates are descriptive only and cannot establish equivalence or comparative effectiveness.

## 4. Materials and Methods

### 4.1. Study Design and Setting

This retrospective observational cohort study was conducted at Memorial Cancer Institute, including Memorial Cancer Institute West and Memorial Cancer Institute East within Memorial Healthcare System in South Florida. Eligible adults with advanced NSCLC harboring actionable oncogenic gene fusions were identified through institutional electronic health records, molecular pathology reports, and oncology registries. Because a separate pre-database screening log was not available, the cohort is described as an eligible analytic cohort rather than as a verifiably consecutive screened population.

The study was approved by the Memorial Healthcare System Institutional Review Board (Protocol No. MHS.2025.175; 13 January 2026) and was conducted in accordance with the Declaration of Helsinki and applicable regulations. The requirement for informed consent was waived because of the retrospective design.

The study is reported in accordance with the Strengthening the Reporting of Observational Studies in Epidemiology (STROBE) statement [26].

Patients diagnosed between 14 May 2014, and 10 October 2025, were eligible. Clinical follow-up continued through the prespecified cutoff of 24 April 2026.

### 4.2. Patient Selection

Eligible patients were identified through institutional molecular testing databases and thoracic oncology registries. Inclusion criteria were: age ≥ 18 years; histologically confirmed lung adenocarcinoma; stage IV or recurrent NSCLC; presence of an actionable ALK, ROS1, RET, NTRK, or NRG1 fusion confirmed by clinically validated molecular testing; receipt of at least one fusion-directed targeted therapy; availability of a documented index-treatment start date; and at least one subsequent clinical or radiographic assessment, documented progression/death event, or censoring date sufficient to calculate follow-up. When multiple sequential fusion-directed therapy records existed for the same patient, only the earliest qualifying fusion-directed therapy was retained as the index observation; later therapy records were treated as subsequent therapy and did not constitute separate patient observations. Patients without molecular confirmation, a fusion-directed index treatment, or the dates required for survival assessment were excluded. One patient with concurrent ALK and EGFR alterations whose recorded treatment was osimertinib was excluded from the analytic cohort because osimertinib is EGFR-directed and did not satisfy the fusion-directed index-treatment definition.

### 4.3. Molecular Testing

Comprehensive molecular profiling was performed as part of routine clinical care using clinically validated assays, including testing available through Tempus (Tempus AI, Inc., Chicago, IL, USA), Caris Life Sciences (Caris Life Sciences, Inc., Irving, TX, USA), Guardant360 (Guardant Health, Inc., Palo Alto, CA, USA), fluorescence in situ hybridization (FISH), and other validated methods as appropriate to specimen availability and testing era. Individual-patient testing-platform, specimen-source (tissue versus plasma), and assay-level DNA/RNA methodology were not systematically retained in the analytic dataset and therefore could not be reconstructed reliably. Testing practices evolved during the 11-year study period; discordant platform-level results could not be systematically adjudicated retrospectively from the locked analytic dataset.

Fusion nomenclature followed current IASLC recommendations. NTRK1, NTRK2, and NTRK3 rearrangements were analyzed collectively as NTRK fusion-positive NSCLC. Among the three unique NTRK-positive patients in the final analytic cohort, one had an NTRK1 alteration specifically documented; the other two were recorded in the source dataset as NTRK1/2/3 without sufficient gene-level detail to assign NTRK1, NTRK2, or NTRK3 individually. The previously included patient with concurrent ALK and EGFR alterations whose recorded therapy was osimertinib was removed from the analytic cohort because osimertinib is an EGFR-directed therapy and was not classified as fusion-directed treatment.

### 4.4. Data Collection

Clinical data were abstracted from the electronic health record using a standardized data collection instrument. Variables included age, sex, smoking history, demographic descriptors, molecular subtype, index targeted therapy, treatment line, dates of progression, death, and last clinical follow-up. The index treatment was defined as the first fusion-directed targeted therapy received for the actionable fusion under study. The index date was the date of initiation of that agent. Subsequent treatment switches, additional fusion-directed therapies, and other systemic therapies did not reset the survival clock; patients continued to be followed for progression and death from the original index date. Demographic fields in the source record included standardized race entries but heterogeneous unstructured descriptors reflecting ethnicity, nationality, geographic origin, and cultural identity. These unstructured descriptors were not treated as mutually comparable ethnic categories and were not used for inferential efficacy analyses. Treatment-naive status was incompletely documented and was excluded from inferential analyses.

### 4.5. Study Outcomes

The primary endpoint was progression-free survival (PFS), defined as the interval from initiation of the index fusion-directed targeted therapy until documented disease progression or death from any cause, whichever occurred first. Overall survival (OS) was defined as the interval from initiation of the same index therapy until death from any cause. Disease progression was determined retrospectively from radiographic imaging reports and treating-oncologist documentation. Formal RECIST 1.1 measurements were not retrospectively reconstructed, and imaging was not centrally reviewed. When radiographic and clinical documentation differed, the progression date recorded by the treating oncology team was used. Imaging was obtained according to routine clinical practice rather than a protocol-mandated schedule. Abstractors were not blinded to molecular subtype. Treatment discontinuation alone was not used as a progression event unless progression was documented clinically or radiographically. Patients without a PFS event were censored at the date of last evaluable clinical follow-up or 24 April 2026, whichever occurred first; patients alive at that time were censored for OS. Secondary endpoints included fusion-subtype distribution, treatment patterns, survival by fusion subtype, and exploratory analyses according to treatment line.

### 4.6. Statistical Analysis

Continuous variables are summarized as medians with ranges or interquartile ranges, and categorical variables as frequencies and percentages. Median follow-up was estimated using the reverse Kaplan-Meier method [27]. PFS and OS were estimated using the Kaplan-Meier method, and survival curves were compared using log-rank tests [28]. Exploratory univariable Cox proportional-hazards models estimated hazard ratios (HRs) and 95% confidence intervals (CIs) for second/third-line versus first-line index therapy [29]. Event counts were reported by treatment-line group. The proportional-hazards assumption was not formally tested in the original analysis; given the small number of events, particularly 15 deaths, the Cox estimates are retained only as descriptive exploratory associations and are not interpreted causally. Broader demographic or multivariable Cox modeling was not performed because key prognostic variables were unavailable, demographic descriptors were inconsistently structured, and the number of events was insufficient for stable adjustment. No correction for multiple comparisons was applied. Analyses were performed using Python version 3.13.5 (Python Software Foundation, Beaverton, OR, USA) and statsmodels version 0.14.6 (statsmodels Developers; open-source software project with no single corporate manufacturer location). All subgroup and regression analyses were considered exploratory and hypothesis-generating.

## 5. Conclusions

Patients with actionable fusion-positive advanced NSCLC experienced durable disease control after genotype-matched targeted therapy in routine clinical practice. This single-system cohort adds a unified descriptive view of precision-oncology implementation across ALK, ROS1, RET, NTRK, and NRG1 fusion-defined disease, including rare fusion subtypes. Because these molecular diseases, targeted agents, treatment generations, and treatment lines are heterogeneous, pooled survival estimates should not be interpreted as general measures of fusion-directed therapy effectiveness or as evidence of equivalent outcomes across subtypes. Larger multicenter studies with standardized clinical covariates, molecular partner/variant annotation, treatment-exposure data, and prespecified comparative methods are needed to address comparative effectiveness and optimal treatment sequencing.

## Figures and Tables

**Table 1:** Baseline Clinicopathologic Characteristics.

Characteristic	Overall (N = 59)
Age at diagnosis, median (range), y	63 (27–90)
Sex	
Female	35 (59.3)
Male	24 (40.7)
Smoking status	
Never	29 (49.2)
Former	29 (49.2)
Unknown	1 (1.7)
Race, as recorded	
White	51 (86.4)
Black	6 (10.2)
Asian	2 (3.4)
Hispanic ethnicity explicitly documented	
Yes	36 (61.0)
No/other unstructured descriptor	23 (39.0)
Histology: adenocarcinoma	59 (100)
Fusion-directed index therapy line	
First	39 (66.1)
Second	15 (25.4)
Third	5 (8.5)
Median follow-up, mo	47.7 (95% CI, 28.6–58.7)

Data are n (%) unless otherwise indicated. Race is presented as recorded in structured fields. The source demographic field also contained heterogeneous unstructured descriptors of ethnicity, nationality, geography, and cultural identity. Only explicit documentation of Hispanic ethnicity is summarized; other descriptors were not treated as mutually comparable ethnic categories and were not used for inferential efficacy analyses.

**Table 2:** Fusion-Specific Targeted Therapy Distribution.

Fusion Subtype	Patients, n (%)	Targeted Therapy	Patients, n
ALK	29 (49.2)	Alectinib	22
		Brigatinib	3
		Crizotinib	2
		Lorlatinib	2
ROS1	14 (23.7)	Entrectinib	7
		Crizotinib	4
		Lorlatinib	2
		Repotrectinib	1
RET	11 (18.6)	Selpercatinib	11
NTRK	3 (5.1)	Larotrectinib	3
NRG1	2 (3.4)	Zenocutuzumab	2

NTRK1, NTRK2, and NTRK3 rearrangements were analyzed collectively as NTRK fusions. Of the three unique NTRK-positive patients, one had NTRK1 specifically documented; the other two were recorded as NTRK1/2/3 without sufficient source detail for gene-level assignment. A sequential repotrectinib treatment record in the NTRK1-positive patient was not counted as a separate patient or index therapy. Osimertinib was not classified as fusion-directed therapy; the patient whose recorded therapy was osimertinib was excluded from the analytic cohort.

**Table 3:** Kaplan-Meier Survival Outcomes.

Population	N	Events/Censored	Median, mo (95% CI)	12-mo, % (95% CI)	24-mo, % (95% CI)	36-mo, % (95% CI)
Overall PFS	59	27/32	44.8 (17.1-NE)	67.8 (53.9–78.4)	59.5 (45.1–71.3)	51.4 (36.4–64.5)
Overall OS	59	15/44	NR (61.8-NE) *	89.4 (77.9–95.1)	81.3 (67.9–89.5)	71.4 (56.1–82.2)
ALK PFS	29	12/17	NR (11.0-NE)	—	—	—
ROS1 PFS	14	9/5	16.6 (7.6-NE)	—	—	—
RET PFS	11	3/8	29.8 (10.5-NE)	—	—	—
ALK OS	29	7/22	NR	—	—	—
ROS1 OS	14	4/10	NR (26.4-NE)	—	—	—
RET OS	11	3/8	61.8 (16.0-NE)	—	—	—

CI, confidence interval; NE, not estimable; NR, not reached; OS, overall survival; PFS, progression-free survival. Events/censored values are reported in that order. * For overall OS, the median was not reached; 61.8 months is the lower confidence-limit crossing and should not be interpreted as an observed median survival time. Subtype estimates for NTRK and NRG1 are not tabulated because of small sample sizes. Late time-point estimates should be interpreted cautiously as the number at risk decreases substantially.

**Table 4:** Exploratory Univariable Cox Regression by Treatment Line.

Outcome	Comparison	HR	95% CI	*p* Value
PFS	Second/third line vs. first line	1.92	0.90–4.10	0.094
OS	Second/third line vs. first line	2.83	1.02–7.83	0.045

CI, confidence interval; HR, hazard ratio; OS, overall survival; PFS, progression-free survival. Models were univariable, unadjusted, and exploratory. Treatment line was not randomized, the proportional-hazards assumption was not formally evaluated in the original analysis, and clinically important confounding could not be addressed; results should not be interpreted causally.

## Data Availability

De-identified data may be available from the corresponding author upon reasonable request and institutional approval.

## References

[1] Bray F., Laversanne M., Sung H., Ferlay J., Siegel R.L., Soerjomataram I., Jemal A. (2024). Global cancer statistics 2022: GLOBOCAN estimates of incidence and mortality worldwide for 36 cancers in 185 countries. CA Cancer J. Clin..

[2] Chen J., Xu C., Lv J., Lu W., Zhang Y., Wang D., Song Y. (2023). Clinical characteristics and targeted therapy of different gene fusions in non-small cell lung cancer: A narrative review. Transl. Lung Cancer Res..

[3] Lindeman N.I., Cagle P.T., Aisner D.L., Arcila M.E., Beasley M.B., Bernicker E.H., Colasacco C., Dacic S., Hirsch F.R., Kerr K. (2018). Updated molecular testing guideline for the selection of lung cancer patients for treatment with targeted tyrosine kinase inhibitors: Guideline from the College of American Pathologists, International Association for the Study of Lung Cancer, and Association for Molecular Pathology. J. Thorac. Oncol..

[4] Penault-Llorca F., Socinski M.A. (2025). Emerging molecular testing paradigms in non-small cell lung cancer management—Current perspectives and recommendations. Oncologist.

[5] Suda K., Mitsudomi T. (2020). Emerging oncogenic fusions other than ALK, ROS1, RET, and NTRK in NSCLC and the role of fusions as resistance mechanisms to targeted therapy. Transl. Lung Cancer Res..

[6] Rosas D., Raez L.E., Russo A., Rolfo C. (2021). Neuregulin 1 gene (NRG1): A potentially new targetable alteration for the treatment of lung cancer. Cancers.

[7] Mok T., Camidge D.R., Gadgeel S.M., Rosell R., Dziadziuszko R., Kim D.-W., Pérol M., Ou S.-H., Ahn J., Shaw A. (2020). Updated overall survival and final progression-free survival data for patients with treatment-naive advanced ALK-positive non-small-cell lung cancer in the ALEX study. Ann. Oncol..

[8] Shaw A.T., Solomon B.J., Felip E., Bauer T., Liu G., Goto Y., Wu Y.-L., Soo R., Mazieres J., Kim D.-W. (2026). Lorlatinib versus crizotinib as first-line treatment for advanced ALK-positive non-small-cell lung cancer: 7-year update from the phase III CROWN study. Ann. Oncol..

[9] Shaw A.T., Riely G.J., Bang Y.J., Kim D.-W., Camidge D., Solomon B., Varella-Garcia M., Iafrate A., Shapiro G., Usari T. (2019). Crizotinib in ROS1-rearranged advanced non-small-cell lung cancer: Updated results, including overall survival, from PROFILE 1001. Ann. Oncol..

[10] Drilon A., Chiu C.H., Fan Y., Cho B.C., Lu S., Ahn M.-J., Krebs M.G., Liu S.V., John T., Otterson G.A. (2022). Long-term efficacy and safety of entrectinib in ROS1 fusion-positive NSCLC. JTO Clin. Res. Rep..

[11] Drilon A., Camidge D.R., Lin J.J., Kim S.-W., Solomon B.J., Dziadziuszko R., Besse B., Goto K., de Langen A.J., Wolf J. (2024). Repotrectinib in ROS1 fusion-positive non-small-cell lung cancer. N. Engl. J. Med..

[12] Drilon A., Subbiah V., Gautschi O., Tomasini P., de Braud F., Solomon B.J., Tan D.S.-W., Alonso G., Wolf J., Park K. (2023). Selpercatinib in patients with RET fusion-positive non-small-cell lung cancer: Updated safety and efficacy from the registrational LIBRETTO-001 phase I/II trial. J. Clin. Oncol..

[13] Zhou C., Solomon B., Loong H.H., Park K., Pérol M., Arriola E., Novello S., Han B., Zhou J., Ardizzoni A. (2023). First-line selpercatinib or chemotherapy and pembrolizumab in RET fusion-positive NSCLC. N. Engl. J. Med..

[14] Griesinger F., Curigliano G., Thomas M., Subbiah V., Baik C., Tan D., Lee D., Misch D., Garralda E., Kim D.-W. (2022). Safety and efficacy of pralsetinib in RET fusion-positive non-small-cell lung cancer including as first-line therapy: Update from the ARROW trial. Ann. Oncol..

[15] Drilon A., Tan D.S.W., Lassen U.N., Leyvraz S., Liu Y., Patel J.D., Rosen L., Solomon B., Norenberg R., Dima L. (2022). Efficacy and safety of larotrectinib in patients with tropomyosin receptor kinase fusion-positive lung cancers. JCO Precis. Oncol..

[16] Schram A.M., Goto K., Kim D.W., Macarulla T., Hollebecque A., O’reilly E.M., Ou S.-H.I., Rodon J., Rha S.Y., Nishino K. (2025). Efficacy of zenocutuzumab in NRG1 fusion-positive cancer. N. Engl. J. Med..

[17] Hess L.M., Han Y., Zhu Y.E., Bhandari N.R., Sireci A. (2021). Characteristics and outcomes of patients with RET-fusion positive non-small lung cancer in real-world practice in the United States. BMC Cancer.

[18] Lu S., Shen L., Wang Q., Chen H., Zhao Y., Li Y., Segall G., Khanal M., Zhang X., Ding D. (2024). Diagnosis, treatment patterns, and outcomes in real-world patients with RET fusion-positive non-small cell lung cancer in China. Adv. Ther..

[19] Overbeck T.R., Reiffert A., Schmitz K., Rittmeyer A., Körber W., Hugo S., Schnalke J., Lukat L., Hugo T., Hinterthaner M. (2023). NTRK gene fusions in non-small-cell lung cancer: Real-world screening data of 1068 unselected patients. Cancers.

[20] Drilon A., Duruisseaux M., Han J.Y., Ito M., Falcon C., Yang S.-R., Murciano-Goroff Y.R., Chen H., Okada M., Molina M.A. (2021). Clinicopathologic features and response to therapy of NRG1 fusion-driven lung cancers: The eNRGy1 global multicenter registry. J. Clin. Oncol..

[21] U.S. Census Bureau QuickFacts: Broward County, Florida. Hispanic or Latino, Percent: 35.0%. https://www.census.gov/quickfacts/fact/table/browardcountyflorida/PST045225.

[22] U.S. Census Bureau QuickFacts: Miami-Dade County, Florida. Hispanic or Latino, Percent: 70.6%. https://www.census.gov/quickfacts/fact/table/miamidadecountyflorida/POP060210.

[23] Chen J., Lu W., Chen M., Cai Z., Zhan P., Liu X., Zhu S., Ye M., Lv T., Lv J. (2024). Efficacy of immunotherapy in patients with oncogene-driven non-small-cell lung cancer: A systematic review and meta-analysis. Ther. Adv. Med. Oncol..

[24] Peng Z., Ding K., Xie M., Xu Y. (2024). Efficacy of immunotherapy in RET fusion-positive NSCLC: A meta-analysis. Heliyon.

[25] Foffano L., Bertoli E., Bortolot M., Torresan S., De Carlo E., Stanzione B., Del Conte A., Puglisi F., Spina M., Bearz A. (2025). Immunotherapy in oncogene-addicted NSCLC: Evidence and therapeutic approaches. Int. J. Mol. Sci..

[26] von Elm E., Altman D.G., Egger M., Pocock S.J., Gotzsche P.C., Vandenbroucke J.P. (2007). The Strengthening the Reporting of Observational Studies in Epidemiology (STROBE) statement: Guidelines for reporting observational studies. Lancet.

[27] Schemper M., Smith T.L. (1996). A note on quantifying follow-up in studies of failure time. Control. Clin. Trials.

[28] Kaplan E.L., Meier P. (1958). Nonparametric estimation from incomplete observations. J. Am. Stat. Assoc..

[29] Cox D.R. (1972). Regression models and life-tables. J. R. Stat. Soc. Ser. B Stat. Methodol..

